# The Role of the Plasminogen Activator Inhibitor 1 ( *PAI1* ) in Ovarian Cancer: Mechanisms and Therapeutic Implications

**DOI:** 10.1055/s-0044-1791734

**Published:** 2024-10-29

**Authors:** Sneha Grace Mathews, R.B. Devi Krishna, Lavanya M., Nandini K., Sanjana Murali, Preet Agarwal, Elizabeth Rani, Andrea Mary F.

**Affiliations:** 1 Department of Human Genetics, Sri Ramachandra Institute of Higher Education and Research, Chennai, Tamil Nadu, India; 2 Department of Gynecology, Sri Ramachandra Institute of Higher Education and Research, Chennai, Tamil Nadu, India; 3 Department of Biotechnology, Hindustan College of Arts and Science, Chennai, Tamil Nadu, India

**Keywords:** *PAI1* gene, OC, metastasis, biomarker, therapeutic target

## Abstract

Ovarian cancer (OC) is one among most significantly fatal gynecological cancers, with late-stage detection and an inadequate prognosis. Plasminogen activator inhibitor-1 ( *PAI1* ) gene anticipates negative outcomes in many different kinds of malignancies. Several research investigations are currently being done to examine the biological role of *PAI1* in OC and the possible benefits of targeted pharmacotherapies. The *PAI1* gene has been linked to the emergence and development of cancer in the ovary. *PAI1* , an inhibitor of serine protease, influences the fibrinolysis and extracellular matrix remodeling, both of which are crucial for tumor expansion and metastatic growth. *PAI1* levels have been discovered to be subsequently more elevated in malignant ovarian tissues than in usual ovarian tissue, demonstrating a potential connection among *PAI1* overexpression and OC development. *PAI1* promotes tumor cell proliferation, movement, and an invasion by influencing the urokinase-plasminogen activators and through interactions with cell surface receptors. In addition, *PAI1* gene contributes to angiogenesis and apoptotic cell death, which contribute to the more hostile phenotypes of OC. The prognostic and therapeutic consequences of focusing on *PAI1* in OC are explored, demonstrating *PAI1* 's potential to be a biomarker and emphasizing for novel treatment approaches. The *PAI1* gene possesses several functions in OC, affecting tumor development, an invasion, and metastatic growth. Comprehending the complicated interactions and mechanisms that regulate *PAI1* in OC may lead to more efficient evaluation and treatment strategies and ultimately enhance patient outcomes.

## Introduction

Ovarian cancer (OC) is a prevalent gynecologic malignancy, ranking third following cervical and uterine cancer. 1 OC makes up 4% of cancers that occur in women and is among the top causes of death from gynecologic cancers. Because early-stage OC is usually asymptomatic, nearly 75% of women present with advanced symptoms when diagnosed. 2 Despite having a lower prevalence than breast cancer, malignancies of the ovary is threefold more fatal, and the mortality rate is anticipated to increase subsequently by the year 2040. 1 Hence, there is a need to establish novel therapeutic strategies for this carcinoma. GLOBOCAN, in 2018 reported a total of 295,414 newly identified cases of OC, resulting in 184,799 fatalities. Around the world, the incidence in year 2018 turned out to be 6.6 per 100,000, with a 3.9 mortality rate. 3 In India, the incidence of OC is roughly around 7.4%. OC contributes to only 3% of all female cancers.

Plasminogen activator inhibitor 1 ( *PAI1* ), also commonly referred to as SERPINE1, is a serine-based inhibitor of protease that serves as a plasma inhibitor for urokinase-plasminogen activators (uPA), thereby regulating fibrinolytic systems and remodeling of tissues. 4 Many malignant cells associated with tumorigenesis expressed *PAI1* . 5 *PAI1* is a multifaceted protein that supervises cell proliferation, migration, adhesion, and transmission of signals. Several investigations demonstrate that high levels of *PAI1* are an indicator of an inadequate clinical outcome in several different kinds of cancers that include gastric, renal, breast, lung, colorectal, and OC implying possible significance of *PAI1* in the advancement of OC. 4 The aim of this review article was to provide a comprehensive and in-depth evaluation on the impacts of *PAI1* gene in OC considering its mechanisms, clinical implication, and potential therapeutic strategies.

## Structure of PAI 1

**Fig. 1:**
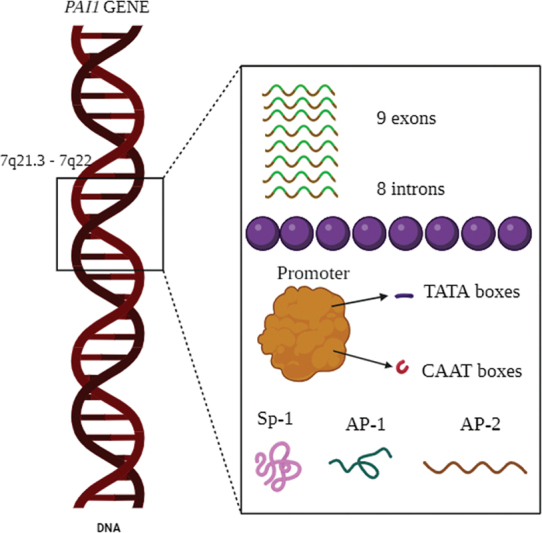
The gene plasminogen activator inhibitor-1 (PAI-1) is found on chromosome 7q22.1. There are nine exons and eight introns in the PAI-1 gene. The noncoding areas of the introns are separated from the coding sequences that generate the protein in the exons. Numerous regulatory elements, including TATA and CAAT boxes, as well as binding domains for transcription factors, including Sp1, AP-1, and AP-2, which control gene expression in regard to different physiological stimuli, are present in the upstream region of the PAI-1 gene.

In humans, *PAI1* gene spans approximately 12,200 base pairs and has up to 9 exons and 8 introns. It is found on chromosome region 7q21.3–7q22 ( Fig. 1 ). Several teams have examined and sequenced the entirety of the *PAI1* gene. Many agents, including dexamethasone, lipopolysaccharide, endotoxin, growth factor, interleukin-1, thrombin, tumor necrosis factors, insulin, and lipoprotein, all promote the synthesis and secretion of *PAI1* . 6 The length of the *PAI1* gene (SERPINE1) is 12.3 kb. Among the nine exons and eight introns, exons code for a 23 amino acid peptide signal and also the mature *PAI1* protein, which is 379 amino acids in length. Furthermore, a mature form consisting of 381 amino acids, which includes two additional amino-terminal (N-terminal) residues, has been recognized, which is presumably the outcome of the cleavage of signal peptidase at an alternate site. Native *PAI1* has a 45-kDa single-chain glycoprotein which lacks in cysteines. 7 The *PAI1* structural gene and its flanking deoxyribonucleic acids (DNAs) consist of two distinct kinds, 12 Alu and 5 Pur of repetitive DNA elements. 8

In *PAI1* , scientists have found three distinct regions that attach to separate protein molecules. The region of *PAI1* responsible for binding to urokinase-plasminogen activator (uPA) is located in the reactive center loop (RCL). When uPA binds to RCL, it causes cleavage in P1-P1' site, resulting in internalization of the N-terminal part in RCL, including P1 region. Another section, located in a flexible area between helices h-D and h-F spanning residues 13-147, binds to somatomedin B region of vitronectin. *PAI1* connects to somatomedin B region of vitronectin, restoring stability, stalling its latent internalization, and enhancing its anti-uPA activity. *PAI1* binds to vitronectin, covering the nearby arginine-glycine-aspartic acid binding site utilized by αv integrins, thus inhibiting cell adhesion. Additionally, a third region within the N-terminal helix h-D, containing the essential Lys 69 residue, attaches on to *PAI1* , either alone or in conjunction with uPA and tissue-type plasminogen activator (tPA), to endocytosis lipoprotein receptor proteins (LRPs) such as LRP-1 and LRP-2. *PAI1* binding to uPA on the surface receptors, facilitated by uPA's attachment to receptor (uPAR), prompts interaction with LRP, leading to internalizing the entire complex inside the cell.

PAI2 is found on chromosome region 18q21.3→18q22.1. *PAI1* and PAI2 are proteins that inhibit uPA and tPA, which are activators of plasminogen. PAI-1 is the main inhibitor of these plasminogen activators, and the placenta secretes PAI-2. PAI-1 is more effective at inhibiting tPA, while PAI-2 is more effective at inhibiting uPA. 9

*PAI1* is a serine protease inhibitor (serpin) that forms a stable acyl-enzyme complex with tPA or uPA, inactivating them and inhibiting plasmin generation. PAI2 is expressed in the placenta and is called placental PAI. It has the same canonical function as PAI1 but has been reported to have different tasks from PAI-1 in cancer.

## Functions of PAI 1

The *PAI1* gene's primary function is to prevent uPA, a metabolic enzyme that breaks down plasminogen to generate plasmin. Plasmin subsequently breaks down the extracellular matrix (ECM), either by itself or with matrix metalloproteinases. *PAI1* attaches to the active site of uPA, hindering the synthesis of plasmin. *PAI1* plays a vital role in the plasminogen-plasmin system. *PAI1* rapidly inhibits tPA, blocking fibrinolysis. Additionally, restricts uPA and engages with various biochemical molecules, including vitronectin and surface receptors. These interactions expand its function to encompass pericellular proteolysis, remodeling of tissue, and cell migration. 10

Reducing *PAI1* levels using very small interfering ribonucleic acid (RNA) leads to notable cell growth inhibition, in OC cells with high *PAI1* expression. Collectively, these findings suggest that *PAI1* facilitates cell development in OC. Surprisingly, ovarian clear cell carcinoma exhibited elevated levels of *PAI1* expression than serous tumors. This observation indicates that reducing *PAI1* can induce cell cycle arrest and apoptosis in OC, pointing to the potential of *PAI1* inhibitors as a novel category of antitumor agents. 4

Motile cells focus uPA on their surface by associating with uPAR, which is abundantly expressed on tumor cells. uPA is recognized as a crucial initiator of plasmin formation during tumor cell invasion and metastasis. Moreover, uPAR interacts with vitronectin, integrins, and the transmembrane receptors, enabling intracellular signaling through effector molecules which affect cell migration, in addition to its uPA binding sites. *PAI1* attaches to vitronectin and hinders its interaction with uPAR and αvβ3 integrin, averting the migration of cells on ECM. Apart from directly inhibiting uPA-mediated plasmin formation, *PAI1* also hampers the activity of the uPA/uPAR complex by promoting endocytosis via LRP-1. This leads to uPA degradation and uPAR recycling. Further, it causes the cell to break away from the ECM. Consequently, *PAI1* was expected to exhibit antitumor effects. Plenty of evidence has been presented to highlight the paradoxical role of *PAI1* in tumor promotion, which can exhibit both proangiogenic and antiapoptotic effects. This functionality is influenced by factors such as the stage of cancer progression, cell type, source (host or tumor), and the relative concentration of *PAI1* . In addition to its connection with vitronectin, *PAI1* has the ability to regulate cell migration through its interaction with the surface receptor LRP-1, which initiates intracellular signaling pathways. 10

*PAI1* plays a crucial part in many different kinds of cellular processes, which include cell migration, tumor metastasis, wound healing, tissue remodeling, and angiogenesis. It has become progressively evident that *PAI1* 's functions go much further than the ability to prevent plasminogen activation from taking place. 11

## Expression and Localization of PAI1

*PAI1* , the protein product of SERPINE1, manages hemostasis, tumor cell migration, ECM remodeling, and invasion. Elevated levels of expression have been linked to an unfavorable prognosis in epithelial OC. 12

The messenger RNA expression of *PAI1* in tumor tissue correlates positively with an unfavorable prognosis for OC patients. To assess *PAI1* 's role in OC cell growth, researchers investigated the effects of inhibiting *PAI1* in cells expressing it. Using small interfering RNA to knock down *PAI1* resulted in notable limitations on cell proliferation, halting of the G2/M phase of the cell cycle and the initiation of intrinsic apoptosis were impacted. Likewise, applying the small molecule *PAI1* inhibitor TM5275 successfully stopped the proliferation of OC cells exhibiting high levels of *PAI1* expression. 4

*PAI1* triggered the formation of cancer-associated macrophages (CAMs), which in turn produced interleukin-8 (IL-8) and C-X-C motif chemokine ligand 5 (CXCL5), promoting the metastasis of OC cells through a feedback mechanism. Activation of the nuclear factor kappa B pathway by *PAI1* in CAMs led to increased expression of downstream targets IL-8 and CXCL5. Furthermore, *PAI1* was associated with peritoneal metastasis in OC patients, indicating a poor prognosis. Both ex vivo and in vivo models demonstrated that knocking out *PAI1* expression significantly reduced metastasis of OC cells. Therefore, targeting *PAI1* could be a promising approach for future therapies aimed at suppressing CAM production and alleviating peritoneal metastasis among OC patients. 13

## Role of PAI1 in Cancer Progression

As per the Cancer Hallmarks Analytics Tool, *PAI1* plays an important role in invasion, metastasis, proliferative signaling, angiogenesis, tumor maintenance, tumor development, and a poor prognosis. Moreover, elevated plasma levels of *PAI1* have been linked to increased susceptibility to atherosclerotic or atherothrombotic complications in certain cancer types. Additionally, *PAI1* may contribute to immunosuppression, which is deemed crucial for rapid tumor advancement. Increasingly compelling evidence indicates that *PAI1* is regulated by numerous microRNAs in tumors, influencing tumor growth and advancement. In addition, different growth factors, some cytokines, and various hormones have all been indicated to increase *PAI1* levels in various malignancies, which can contribute to tumor progression for activating key signaling pathways. 14

The multifaceted function of *PAI1* in cancer development can be explained by its complex structure and multiple roles that extend beyond antifibrinolytic and antiplasminogen activation. Nevertheless, in spite of many pieces of evidence supporting *PAI1* 's protumorigenic role in malignancies and the eventual discovery of multiple inhibitors, targeting *PAI1* remains elusive. 9

The *PAI1* gene encodes a protein that plays a role in regulating fibrinolysis, the process by which blood clots are broken down. This protein inhibits tPA and uPA, which are involved in the breakdown of fibrin, a key component of blood clots. Several studies have suggested that the *PAI1* gene and its protein product might be involved in angiogenesis, invasion, metastasis, and tumor-promoting inflammation ( Table 1 ). 15

**Table 1:** Several roles or functions of the PAI1 gene in cancer advancement

Cancer hallmarks	Effects	PAI1 region	Mechanisms
Sustaining proliferative signals	Increases the G0/G1 to S phase transition	Undetermined	Increased expression of cyclin E, forming complexes with CDK while reducing levels of p53, p21, and p27
	Controls adherence of the cell	Bind to vitronectin	Enhances attachment to fibronectin, leading to subsequent cell proliferation
	Supports senescence and dormancy	uPA binding (Reactive Center Loop)	Modifying the ERK/p38 ratio through inhibition of uPA/uPAR
	Prevention of fibrinolysis.	Binding of uPA to the reactive center loop	Stimulates PAR activation through thrombin, leading to an upregulation of PAI1
	Hinders EGFR signaling	uPA/uPAR/PAI1 complexes	Inhibits uPA/uPAR interactions with EGFR 4 5 12
Preventing cell death	Reduces intrinsic apoptosis	Binding of uPA to the reactive center loop	Intracellular caspase 3 inhibition
	Causes anoikis	Vitronectin binding domain	Prevents adhesion of cells to vitronectin, increases migration of cells and fibronectin adherence (double edge)
	Decreases programmed cell death	Binding of uPA to the reactive center loop	Decreased cleavage and release of FasL by plasmin curbs FasL-triggered apoptosis in tumor cells
	Provides protection against apoptosis	Binding to LRP-1	Encourages cJun/ERK activation and increases the expression of the Bcl-2 and BclXL 4 5 12
Angiogenesis	Enhances endothelial cell (EC) viability	Binding of uPA to the reactive center loop	Reduction in FasL cleavage and shedding due to plasmin inhibits FasL-induced apoptosis in EC
	Blocks EC attachment to vitronectin	Region involved in binding to vitronectin	Induction of EC migration from vitronectin to fibronectin
	Enhances EC organization in fibrin matrix	Binding of uPA to the reactive center loop	Enhances EC attachment to fibrin and production of IL-8
Invasion and metastasis	Regulates migration	uPA binding and vitronectin binding	Encourages cell release from vitronectin and inhibits excessive degradation of ECM proteins
	Increase in vivo metastasis	Undetermined	Not defined
	Decrease in vivo metastasis	Undetermined	Not defined
	Inhibition of fibrinolysis	Binding of uPA to the reactive center loop	Facilitates NET 4 5 12
Tumor promoting inflammation	Increase monocyte/macrophage migration	Binding to LRP	Brings monocytes to the tumor location
	Enhances macrophage M2 polarization	Binding of uPA to the reactive center loop	Triggers p38/IL-6/NFkB/STAT3 activation 4 5 12

Abbreviations: CDK, cyclin-dependent kinase; ECM, extracellular matrix; EGFR, epidermal growth factor receptor; ERK, extracellular signal-regulated kinase; IL-8, interleukin-8; LRP-1, lipoprotein receptor protein-1; NET, neutrophil extracellular trap; NFkB, nuclear factor kappa B; PAI1, plasminogen activator inhibitor-1; PAR, protease-activated receptor; STAT3, signal transducer and activator of transcription 3; uPA, urokinase-plasminogen activator; uPAR, urokinase-plasminogen activator receptor.

*Angiogenesis* : *PAI1* is involved in regulating angiogenesis, the formation of new blood vessels. In the context of OC, *PAI1* affect the angiogenic process, which is crucial for tumor growth and metastasis.

*Tumorprogression* : Elevated levels of *PAI1* have been associated with increased tumor progression in various cancers, including OC. High *PAI1* expression can contribute to a more aggressive cancer phenotype by promoting ECM remodeling and facilitating tumor invasion. 4 16

*Invasion andmetastasis* : *PAI1* plays a crucial role in inhibiting the activity of plasminogen activators like tPA and uPA. These activators are involved in the breakdown of fibrin and the ECM. By inhibiting these processes, *PAI1* can contribute to a more stable ECM, which can support the invasion and spread of cancer cells. In OC, high *PAI1* levels have been associated with increased invasive potential and metastatic spread of cancer cells into surrounding tissues and potentially into distant sites. 9 17

Overall, while the *PAI1* gene is not the sole determinant of OC development, its expression and regulation can have significant implications for cancer progression. Elevated *PAI1* levels are often associated with more aggressive forms of OC and poorer prognosis. Continued research is essential to fully understand these mechanisms and to explore *PAI1* as a potential biomarker and therapeutic target in OC. 16

## Mechanism of the *PAI1* Gene in Ovarian Malignancies

**Fig. 2:**
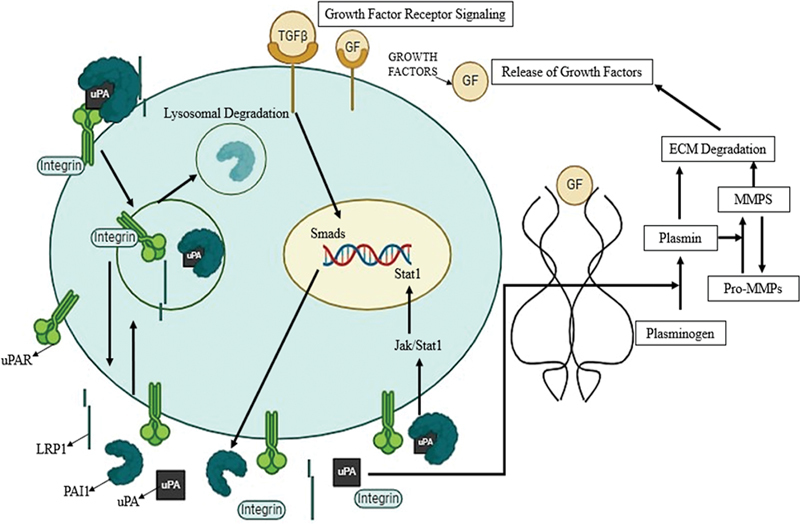
Plasminogen activator inhibitor-1 (PAI1)-mediated signaling pathways play a role in regulating various cellular processes, particularly those related to tumorigenesis and progression. The integrin complex including urokinase-plasminogen activator (uPA), uPA receptor (uPAR), and PAI1 triggers endocytosis via lipoprotein receptor protein (LRP)-1 and very-low-density lipoprotein receptor (VLDL-R). The process finally results in the recovery of integrin, uPAR, and LRP-1 to the plasma membrane and the lysosomal destruction of the uPA and PAI1 complexes. This activation eventually promotes the invasion and proliferation of cancer cells by activating the extracellular signal-regulated kinase (ERK) pathway. Furthermore, this promotes matrix metalloproteinase's (MMP's) gene expression patterns, which aid in the invasion of cancer cells.

*PAI1* , commonly referred to as *PAI1* , is a vital physiological inhibitor for endogenous plasminogen activators. It restricts fibrin breakdown, facilitates the deposit of fibrin on the walls of blood vessels, and induces proliferation in smooth muscle cell. 18 *PAI1* is a highly effective inhibitor of the plasminogen activators, which includes tPA and uPA. 15 Activation of plasmin is enabled by PA and finely regulated by SERPINE1 through many extracellular and intracellular pathways ( Fig. 2 ). The binding of *PAI1* to the uPA, uPAR, and integrin complex initiates endocytosis by members of the low-density lipoprotein-receptor gene family, such as LRP-1 and very-low-density lipoprotein receptor. This process ultimately culminates in lysosomal degradation of uPA and *PAI1* complexes and the retrieval of integrins, uPAR, and LRP-1 to plasma membrane. The above mechanism facilitates equilibrium in both localized adherence of cell and ECM turnovers. Pericellular active, concealed, or cleaved *PAI1* can attach specifically to *LRP-1* , causing the phosphorylation of tyrosine of the cytoplasmic tail sequence and stimulating Jak/Stat1 pathway. Translocation of Stat1, nuclear material, causes the de novo synthesis of *PAI1* . uPAR, integrins, and *LRP-1* could be targeted at motile cell's leading edge while during the process of recycling in invasive cells. In the invasive front of the malignant cells, focal proteolysis processes change the balance between maintaining the ECM and its degradation, releasing growth factors and cytokines embedded within the matrix which result in additional transcriptional upregulation of the *PAI1* gene. Higher *PAI1* levels, which are usually observed in the environment surrounding extremely invasive cells, result in increased proteinase manufacturing through PAs and a beneficial impact on the migration of cells. 19

## Influence of PAI1 Gene in OC Development

The influence of the *PAI1* gene on OC is multifaceted, affecting tumor development, progression, and potentially contributing to its causation.

*Inhibition of ECM degradation* : *PAI1* inhibits plasminogen activators (tPA and uPA) that are involved in the breakdown of fibrin and ECM components. By preventing ECM degradation, *PAI1* can create a more favorable environment for ovarian tumor cells. This stabilization of the ECM can support OC cell growth, survival, and invasion into surrounding tissues. 4 20

*Increased invasion potential* : High levels of *PAI1* have been associated with enhanced tumor invasion and metastatic potential. *PAI1* 's inhibition of ECM degradation can facilitate the invasion of OC cells into surrounding tissues and promote their spread to distant sites. 13

*Impact on blood vessel formation* : *PAI1* can influence angiogenesis, the formation of new blood vessels necessary for tumor growth. By affecting ECM remodeling and stability, PAI1 can alter the angiogenic process, potentially supporting tumor expansion and metastasis.

*OC cell dynamics* : Elevated *PAI1* levels can affect cancer cell behavior, including cell adhesion, migration, and proliferation. These effects contribute to the aggressive nature of OC and its progression. 4 14

*Influence on tumor microenvironment* : The *PAI1* gene can affect the tumor microenvironment, including the interaction between tumor cells and surrounding epithelial and stromal cells. By modifying the ECM and influencing cellular interactions, *PAI1* can contribute to the development and progression of OC. 4 12 21

*Inflammation* : Chronic inflammation is a known risk factor for cancer. *PAI1* may influence inflammatory responses in the tumor microenvironment, potentially contributing to OC initiation and progression.

## Clinical Implication and Potential Therapeutic Practice

The *PAI1* gene has recently been identified in various kinds of cancers, including OCs, and targeting this gene's involvement in OC could be a promising therapeutic approach.

The plasminogen activation system as a whole, considered to be necessary for the growth, development, invasion, and cancer metastasis, is modulated by *PAI1* . In cases involving OC, higher aggressiveness of the tumor and chances of metastasis have been correlated with increased levels of *PAI1* . According to this, *PAI1* could potentially be used as a prognostic indicator to estimate the chance of disease progression and metastasis. Furthermore, chemoresistance in several kinds of cancers, including OC, has been related to high levels of *PAI1* . Chemoresistance poses a serious threat to OC treatment, increasing the risks, chances of treatment failure, and disease recurrence.

By increasing cancer cell survival and inhibiting the therapeutic effects of some chemotherapeutic drugs, *PAI1* can aid in chemoresistance. In order to get past this treatment barrier, it could potentially be possible to find novel therapeutic targets by comprehending the role of *PAI1* in chemoresistance. It has been reported that the expression levels of *PAI1* could be used as prognostic indicators for OC. Studies have revealed that higher levels of *PAI1* are connected with adverse outcomes such as reduced overall survival rates and progression-free survival. Therefore, the expression of *PAI1* may be beneficial in anticipating patient outcomes and coordinating treatment choices, like selecting more potent treatment options or signing up for clinical trials. Therapeutically targeting *PAI1* might constitute a promising strategy for treating OC. The inhibition of tumor growth, metastasis, and chemoresistance through strategies targeting *PAI1* expression or activity could enhance treatment outcomes for patients with OC. 20 22 23

Several preclinical investigations have examined the effectiveness of *PAI1* -targeted therapies in OC models, indicating the potential for therapy of such a strategy. Keeping track of *PAI1* levels throughout treatment could prove to be useful as a biomarker, to determine the response to therapy and progression of the disease in OC patients. Modifications in *PAI1* expression levels following the commencement of treatment could offer valuable insights into therapy's effectiveness and assist in treatment modifications as needed. 13

Potential therapeutic practices involving *PAI1* includes *PAI1* inhibition techniques, combination therapy, gene silencing, immunotherapy, and personalized medicine.

➢ *PAI1 inhibition* : Small molecular inhibitors or monoclonal antibodies (mAbs) that are specifically against *PAI1* could one day be invented for minimizing its level of expression or function within OC cells. By restricting *PAI1* , inhibitors like these may help to prevent progression of the tumor, metastasis, and chemoresistance, potentially enhancing the outcome of treatment for OC patients. Preclinical investigations with *PAI1* inhibitors have shown promising results in restricting OC development and metastasis. 4 24
➢ *Combination therapy* : *PAI1* inhibitors can be incorporated into conventional chemotherapy drugs to enhance their effectiveness in dealing with OC. The combination of *PAI1* inhibitors with chemotherapy has the potential to reduce chemoresistance and boost response to treatment by targeting several tumor progression and metastasis pathways. Preclinical investigations have demonstrated beneficial interactions between the *PAI1* inhibitors and the chemotherapy agents, demonstrating the possibility of combination therapy strategies. 21
➢ *Gene silencing* : RNA interference or gene editing methods like CRISPR/Cas9 could potentially be utilized to silence or to knock out the *PAI1* gene within cells with OC. These strategies, which suppress *PAI1* expression, could hinder tumor growth, metastasis, and chemoresistance, leading to improved therapeutic outcomes. Gene silencing methods focused on *PAI1* have displayed assurance in preclinical research as a potential approach for treating OC. 25 26
➢ *Immunotherapy* : *PAI1* could potentially be targeted by employing immunotherapy, which encompasses mAbs or chimeric antigen receptor T-cell therapy. Immunotherapy, which promotes the body's immune system to detect and attack OC cells that express *PAI1* , might provide a novel treatment option to patients with *PAI1* -positive cancers. Immunotherapeutic targeting of the *PAI1* gene has demonstrated promising outcomes in preclinical research and requires additional research in clinical investigations for OC. 27 28
➢ *Personalized medicine* : Patient differentiation according to *PAI1* expression levels and other genetic and molecular characteristics might help recognize OC patients who can benefit from *PAI1* -targeted treatment options. Personalized therapy approaches customized for each patient's cancer profile might improve the effectiveness of therapy while minimizing negative effects. Integrating *PAI1* gene expression profiling into medical decision-making algorithms might enhance the choice of treatment and patient outcomes in OC. 27 29 30

## Challenges in Future Directions

OC is an extremely complicated disease that includes multiple molecular subtypes and genetic mutations. Comprehending the complex interactions of the *PAI1* alongside other molecular pathways associated with OC metastasis, progression, and chemoresistance is crucial in establishing efficient therapeutic strategies. Discovering the complex nature of tumor cell biology and determining the important regulators of *PAI1* expression and activity within OC cells are major obstacles for future research. While *PAI1* has demonstrated potential as a prognostic marker and target for therapy in OC, more proof studies are required to validate its clinical relevance. A large-scale clinical trial is required for verifying the prognostic significance of *PAI1* expression levels and to establish their predictive value for response to treatment and outcome among patients. 31 32 Established techniques for evaluating *PAI1* expression, alongside reliable biomarker validation protocols, must be developed to translate study results into clinical application. Chemoresistance remains an obstacle in the curative process of OC, which is limiting effectiveness of conventional chemotherapy regimens. *PAI1* has been associated with the emergence of chemoresistance via several processes, which includes apoptosis restriction and cancer cell survival advancement. In order to overcome resistance to treatment by focusing on *PAI1* along with other related pathways, innovative and beneficial therapeutic methods are necessary, which might include combination therapies and precision medical strategies customized to individual profiles of patients. 33 34 35

Establishing *PAI1* -targeted therapies using optimal effectiveness and safety profiles poses several issues. Selecting the most effective therapeutic agents, such as small molecular inhibitors, gene editing technologies, and mAbs necessitates cautious consideration of target specificity, delivery of drugs, and potential off-target consequences. Optimizing treatment strategies, dosage schedules, and combination therapies to optimize the effectiveness of therapy and minimize toxicity is crucial to enhancing outcomes for patients with OC. 12 21 36 Interpreting preclinical findings on *PAI1* , into clinically pertinent treatment strategies is a significant challenge in OC research. In order to bridge the gap between benchtop innovations and bedside implementation, clinical investigators and health care professionals have to collaborate on a multifaceted level. 37 38 Developing the infrastructure for translational investigations, such as patient-derived malignancy models and biomarker validation research, is essential for speeding up the clinical implementation of *PAI1* -targeted treatment options in OC. 39 40

## Conclusion

Genetic variations in the *PAI1* gene are currently been investigated for possible significance in OC progression, metastasis, chemotherapy resistance, and patient outcomes. Comprehending its medical implications could result in the development of novel prognostic markers and therapeutic approaches for patients with OC, thereby enhancing the management and treatment outcomes. Ultimately, targeting the *PAI1* gene is an exciting approach for therapy, with the potential for improving the effectiveness of treatment, surpassing chemoresistance, while enhancing patient outcomes. In order to tackle the challenges, scientific communities, medical practitioners, industrial partners, and patient advocates must work together to advance our understanding of the *PAI1* gene's role in OC and interpret this knowledge into effective clinical treatments that improve patient outcomes. The potential therapeutic implications of targeting *PAI1* in OC give hope for innovative treatment strategies that could eventually transform OC management and care. Further investigations are needed to fully understand these associations and apply them in clinical practice to ameliorate OC management.
